# Exploring How UK Adults' Attachment Style in Romantic Relationships Affects Engagement in Controlling Behaviours

**DOI:** 10.3389/fpsyg.2021.649868

**Published:** 2021-06-30

**Authors:** Molly C. Gilbert, Robert Blakey

**Affiliations:** Department of Psychology, University of Bath, Bath, United Kingdom

**Keywords:** disorganised attachment, controlling-punitive, controlling-caregiving, adult attachment, insecure attachment, controlling behaviours, relationships, caregiving

## Abstract

Copious studies have identified a link between disorganised attachment and engagement in controlling caregiving or controlling punitive behaviours. Studies have suggested that consistently engaging in these behaviours can cause difficulties within relationships and contribute to the development of a personality disorder. Most of the literature thus far has focused on engagement in controlling behaviours by children with a disorganised attachment style, despite there being theoretical grounds to suggest they may also be used by adults and across all types of insecure attachment. This study aimed to address these gaps by looking at adult attachment style and engagement in controlling behaviours in romantic relationships, across all insecure attachment styles; avoidant, anxious and disorganised. The current study recruited a non-clinical sample; specifically, 149 English-speaking adults, living in the UK, between the ages of 18 and 77 years old (*M* = 34.28, *SD* = 14.90). The participants answered an anonymous online questionnaire containing four self-report measures which assessed the participants' attachment security and organisation, caregiving style and engagement in punitive behaviours. The results indicated that participants who scored higher in disorganised attachment were more likely to use controlling punitive behaviours in their romantic relationships. Moreover, participants who reported a more insecure-anxious attachment style were more likely to use compulsive caregiving behaviours in their romantic relationships. In contrast, participants who reported a higher insecure avoidant attachment style were less likely to use compulsive caregiving behaviours in their romantic relationships. These results have implications for adult attachment theory and aid the understanding of some of the behaviours that can be harmful within romantic relationships. The findings could be used to help at-risk individuals develop healthy interpersonal relationship going forward.

## Introduction

Several developmental theories illustrate the impact of early life experiences on an infant's development. One such theory, attachment theory, considers the attachment between infants and their primary caregivers to have a substantial effect on the infant's development. This theory defines attachment as a lasting experience of psychological connectedness between human beings (Bowlby, 1969). Bowlby suggested that infants are born with a pre-disposition to form attachments with their caregivers; they seek close proximity to a caregiver who can provide security and safety thus enhancing their chance of survival. Attachment theory provides the bedrock for the current study, which assessed the attachments made by adults in their romantic relationships. Bowlby (1969) theorised that adult attachments are greatly influenced by the first attachments made as an infant. Therefore, this paper begins by discussing the distinctions between the various types of attachment that infants can make to their caregivers.

Ainsworth et al. (1969) constructed the Strange Situation Classification to assess the type of attachment that infants made to their caregivers. Based on this research, three attachment styles were identified: secure, insecure avoidant and insecure ambivalent (anxious) (Ainsworth and Bell, 1970). Disorganised attachment style was later added as a fourth attachment style (Main and Solomon, 1990). These four attachment styles are believed to play an important role not only in childhood but also in adulthood (Mikulincer and Shaver, 2007); hence the current study also used these categorisations of attachment style. Various methods have been used to assess adult attachment style. The Adult Attachment Interview has been widely used in existing literatures; however, this method involves lengthy interviews and a time-consuming coding process (Main et al., 1985; Scharfe, 2016). Social psychological studies have tended towards the use of self-report measures of adult attachment, such as the Experiences in Close Relationships scale and Adult Disorganised Attachment scale which have been found to have high internal consistency (Main et al., 1985; Brennan et al., 1998; Paetzold et al., 2015). However, these measures are specifically designed to measure either disorganised or insecure attachment and there is not currently a reliable self-report scale to measure the four categories of adult attachment (Scharfe, 2016).

In the Strange Situation Classification study, most of the children were classified as securely attached; they used their attachment figure as a safe base to explore from and were easily soothed by their attachment figure when distressed (Main and Cassidy, 1988). Children classified as insecure avoidant were very independent from their attachment figure, who was often unavailable and insensitive to their needs, and did not seek them when distressed (Behrens et al., 2007). Those with insecure anxious attachments often presented as dependent and clingy; they did not explore their environment and were not easily comforted by their caregiver. Finally, infants with a disorganised attachment style often had caregivers who were either frightened or frightening when the child was distressed, leaving them confused and inconsistently soothed (Lyons-Ruth et al., 1997). When the caregiver is a source of fear, the child faces an internal conflict between their innate need to seek safety and comfort and their defence system which encourages them to avoid the caregiver (Slade, 2014).

Attachment theory states that through the close early relationships with caregivers, infants develop an internal working model from which they make sense of themselves, their environment and other people's ability to provide care (Bowlby, 1973). Research has shown that attachment strategies learnt in infancy tend to remain stable across the lifespan, however in adulthood the primary attachment figure in adulthood is often close, romantic partners (Paetzold et al., 2015; De Carli et al., 2016). An individual's internal working model can affect how they form relationships throughout their life; for example, parents often treat their children in the way they themselves were treated as children (Hazan and Shaver, 1987; Feeney and Noller, 1990; Kirkpatrick and Davis, 1994; Howe, 1995). Securely attached children are likely to develop a positive internal working model of themselves as worthy of respect and loveable, and view others as trustworthy (Jacobsen and Hofmann, 1997). In contrast, children with attachment insecurity, particularly disorganised attachment, have an increased risk of developing a negative internal working model; which may contribute to social and behavioural problems, and psychopathology, such as a personality disorder (Bretherton and Munholland, 2008; McCarthy and Maughan, 2010). However, this link has not been widely explored in relation to adult attachment and caregiving behaviours towards a romantic partner, which the current study aims to rectify.

Personality disorders are deeply ingrained patterns of behaviour that deviate considerably from cultural expectations and can be highly problematic for individuals in their interpersonal relationships or functioning in society (American Psychiatric Association, 2013; Ekselius, 2018). Disorganised attachment style has been associated with many personality disorders, however, particularly Borderline Personality Disorder, which is characterised by instability in interpersonal relationships, as well as difficulties with self-identity, impulsivity and affect regulation (Fonagy, 1999; Leichsenring et al., 2011; American Psychiatric Association, 2013; Liotti, 2013). Insecure avoidant individuals are more likely to develop Cluster A personality disorders, such as Schizoid Personality Disorder, which often involve difficulty trusting others and maintaining close relationships (Troy and Sroufe, 1987; Sinha and Sharan, 2007). In contrast, those with an insecure anxious attachment style are more likely to develop personality disorders which involve neediness and dependency, such as Histrionic or Dependent Personality Disorder (Sinha and Sharan, 2007). Hence there are both theoretical reasons and some empirical research to suggest links between specific attachment styles and personality disorders. The current study cannot explain the development of personality disorders in adulthood as this pathway is complex and not yet fully understood. However, disturbances in interpersonal relationships are commonly found across a range of personality disorders and this study aims to explore the link between adult attachment and some behaviours which can impact the formation and maintenance of relationships.

Studies have explored the mechanisms linking attachment styles and personality disorders, to understand how interpersonal problems during development contribute to personality pathology (Lyddon and Sherry, 2001; Crawford et al., 2007). A wealth of literature has illustrated that children who are insecurely attached, particularly disorganised, are more likely to engage in rigid controlling behaviours in relationships with their attachment figures to help regulate their insecurity by making the other person subordinate or dependent (Hollidge and Hollidge, 2016). This study highlights that without the positive internal working model that attachment security brings, insecure people rely heavily on controlling behaviours to elicit a constant supply of external sourced self-esteem (Bowlby, 1973).

These controlling behaviours involve inborn tendencies like caregiving or competitive aggression (Liotti, 2017). Caregiving is illustrated by a tendency to nurture, whereas the goal of the competitive system is to control others by asserting dominance in social interactions (Liotti, 2017). Competitive aggression is generally termed controlling punitive behaviour within the literature and hence this term will be used by the current study. In children, controlling punitive behaviour often leads to bossy and aggressive behaviour toward the caregiver (Lyons-Ruth and Jacobvitz, 2008). Controlling punitive behaviour has been associated with externalising disorders, which generally involve problematic behaviour, aggression and impulsivity, and is more likely to occur when the infant's caregivers adopt a submissive attitude towards the infant (Hesse et al., 2003; Moss et al., 2004; Samek and Hicks, 2014).

Controlling caregiving behaviour, on the other hand, is frequently associated with a role reversal in caregiving, called inverted attachment, where children care for their vulnerable or helpless guardians instead of their guardians caring for them (Hesse et al., 2003; Liotti, 2011; Solomon and George, 2011; Lecompte and Moss, 2014). Controlling caregiving behaviour has been associated with internalising disorders such as anxiety and depression (Moss et al., 2004). The absence of attachment security can lead people to regulate their negative working model by engaging in controlling behaviours, either punitive or caregiving.

In children, there is a clear link between attachment style and engagement in controlling behaviours. A wealth of attachment literature has shown that disorganised infants have an increased tendency to control their guardians' attention using either punitive or caregiving behaviours (Main and Cassidy, 1988; Cassidy and Marvin, 1992; van IJzendoorn et al., 1995; Green and Goldwyn, 2002; Lyons-Ruth, 2007; Liotti, 2011, 2017). It has been suggested that these controlling behaviours function to protect the child from their insecure attachment style, to help them to cope and organise their social interactions (Liotti, 2017).

Research suggests that whilst the controlling behaviours are in place, insecure attachment styles do not predict psychopathology in childhood (Liotti, 2011). However, there is a debate in the literature regarding whether insecure attachment styles predict engagement in controlling behaviours and psychopathology long-term. A longitudinal study concluded that engagement in controlling behaviours is consolidated by 5 years old, with other researchers also proposing that controlling behaviours stabilise over time and henceforth contribute to the development of a personality disorder (Brennan and Shaver, 1998; Moss et al., 2005; Solomon, 2018). Whilst previous studies seem to indicate that the behaviours associated with insecure, particularly disorganised, attachment in childhood remain fairly stable into adulthood, very few have directly explored attachment style and engagement in controlling behaviours in adulthood (Green and Goldwyn, 2002). The current study aimed to address this gap in the literature by exploring the link between attachment style and controlling behaviours in adult relationships.

Beeney et al. (2017) explored the association between disorganised adult attachment and personality disorder symptom severity. They concluded that disorganised attachment styles are linked to functional impairment in work, social and romantic functioning, identity and mentalisation. However, it was an exploratory study containing biases by the clinicians regarding the attachment ratings assigned due to a knowledge of diagnosis and severity. Whilst the association between attachment style and symptoms of personality disorders were considered, the study was exploratory and unable to draw a clear and direct pathway between disorganised attachment and the development of a personality disorder. Another recent study reviewed the state of research on the associations between romantic attachment insecurity and Borderline Personality Disorder (BPD) traits to help understand BPD symptoms further such as disordered romantic functioning (Smith and South, 2020). Deficits in interpersonal relationships which occur within personality psychopathology are widely theorised about, with many studies focussing on attachment as an explanation. However, the pathway from attachment to a personality diagnosis is not yet clear. For example, there is a debate in the literature regarding whether BPD symptoms are most consistent with insecure anxious or insecure avoidant attachment (Levy et al., 2015). Whilst others believe that there are strong links between disorganised attachment and BPD (Main et al., 1985; Selby et al., 2008; Beeney et al., 2017). Smith and South (2020) sought to summarise the often-conflicting literature surrounding the link between BPD traits and attachment style focussing on adult attachment in the context of romantic relationships. Their findings indicated that both forms of attachment insecurity were correlated with BPD traits, however a clear pathway was not determined. Similarly, the current study also focussed on romantic attachment and sought to explore the associations between insecure attachment styles and behaviours that can affect the development and maintenance of healthy interpersonal relationships. Whilst the existing literature contains much debate and uncertainties surrounding the pathway from attachment insecurity and personality psychopathology, the current study aimed to improve the understanding of some of the controlling and caregiving behaviours that are thought to contribute to the common symptom of various personality disorders, unstable relationships.

The current study distinguished from previous research in three respects. First, whereas previous research has documented the link between disorganised attachment style and controlling behaviours in childhood (Liotti, 2011), this study examined the same link in adulthood. Second, whereas previous research focuses on the link in relation to disorganised attachment style (Lyons-Ruth, 2007), the current study examined the full range of insecure attachment styles; disorganised, avoidant and anxious. This is important given the wealth of evidence highlighting the link between insecure attachment, both avoidant and anxious, and personality disorders (Sinha and Sharan, 2007). Third, whereas previous research has focused on the attachment between the child and caregiver (Liotti, 2017), the current study examined the attachment between two adults in a romantic relationship. This focus was considered to have applied implications given that personality disorders are frequently associated with dysfunction in romantic relationships (Selby et al., 2008; Ekselius, 2018). Additionally, the focus of exploring controlling and caregiving behaviours to help understand some of the behaviours that can impact relationships, was particularly relevant considering the recent Covid-19 pandemic. The pandemic, lockdowns and socially distanced measures that have been implemented have greatly altered people's lives and social interactions (Pietromonaco and Overall, 2020). Research has shown that Covid-related stresses can be harmful to romantic relationships and undermine relationship quality and that these harmful effects are likely to be exacerbated by individual vulnerabilities such as attachment (Pietromonaco and Overall, 2020). Thus, illustrating the importance of the current study's focus, as insecurely attached individuals are more vulnerable to relationship breakdown and challenges in the event of external stressors such as the pandemic. It is necessary to gain a better understanding of the association between attachment and some of the behaviours that can be harmful within relationships, to help at-risk individuals to develop and maintain healthy relationships in the future.

To investigate the link between attachment style and controlling behaviours in adult romantic relationships the following hypotheses were proposed. Firstly, participants with higher levels of disorganised attachment would be more likely to engage in controlling caregiving behaviours in romantic relationships. This hypothesis was proposed from evidence that disorganised children tend to engage in controlling caregiving behaviours towards their caregivers (Liotti, 2011, 2017). These behaviours are common in children who had a frightened or helpless guardian for whom the child provided care (inverted attachment) (Solomon and George, 2011). Studies have indicated that engagement in controlling caregiving behaviours may be an attempt to nurture the vulnerable guardian to re-establish them as the protective caregiver (George and Solomon, 2008). Therefore, disorganised individuals with an inverted attachment style, may be more likely to engage in controlling caregiving behaviours (as opposed to punitive). The current study aimed to replicate and extend this finding to the novel context of adult romantic relationships, where the caregiving system is two-sided. Romantic partners seek care from and provide care to one another (Mikulincer and Shaver, 2007). Functional caregiving within adult romantic relationships is characterised by an awareness of a partners attachment behaviours, and an ability to respond with empathy to their distress signals (Mikulincer and Shaver, 2005). However, when this system is dysfunctional individuals may be either over or under sensitive to a partner's needs; both of which can have ramifications on the relationship quality (Gabbay and Lafontaine, 2017).

Secondly, it was hypothesised that participants with higher levels of disorganised attachment would be more likely to engage in controlling punitive behaviours in romantic relationships. This hypothesis drew on previous studies' conclusions that disorganised children were more likely to engage in controlling punitive behaviours, to organise their interactions with their caregivers (Lyons-Ruth, 2007; Liotti, 2011, 2017). Using such behaviours demand the caregiver's attention and can help to mask pain through anger and detachment. Disorganised individuals may choose to engage in authoritarian, controlling punitive behaviours to become dominant and protect themselves (Forrest, 2008; Liotti, 2011). The purpose of this hypothesis was to explore whether the engagement in controlling punitive behaviours persists into adulthood in disorganised individuals.

The third hypothesis was; participants with a higher insecure avoidant attachment style would be more likely to engage in controlling punitive behaviours in romantic relationships. This hypothesis was based on the previous findings that individuals with an insecure avoidant attachment would use more competitive social dominance seeking behaviour and aggressive antisocial behaviour (Troy and Sroufe, 1987; Westen et al., 2006; Yip et al., 2018). Likewise controlling punitive behaviours are characterised by antisocial behaviour such as hostility or anger, used in an effort to compete for social dominance (Bureau et al., 2009; Hawley et al., 2009). For this reason, this study aimed to explore whether there was an association between the two.

The final hypothesis in this study was; participants with a higher insecure anxious attachment style would be more likely to engage in compulsive caregiving behaviour in romantic relationships. The rationale behind this hypothesis considered the previously identified association between insecure anxious attachment and histrionic or dependent personality disorder which are characterised by neediness and overdependence on others (Sinha and Sharan, 2007). Research has shown that adults' attachment patterns are closely linked to their caregiving responses towards others (Julal and Carnelley, 2012). Compulsive caregiving is defined as an over-involvement in a partner's problem-solving efforts and is often characterised by a persistent need to ignore own needs in favour of focussing on the needs of others (Kunce and Shaver, 1994; Feeney et al., 2001). Such individuals are very dependent on their partner for fulfilment which is also common in dependent and histrionic personality disorders (Beeney et al., 2017). The personality disorder tendency in insecure anxious individuals suggests that they may be more likely to engage in compulsive caregiving behaviours within their romantic relationships.

## Materials and Methods

### Participants

Any English-speaking adult, over the age of 18, living in the UK, was eligible to partake in this study. In total, 188 adults were recruited; however due to attrition, only 149 participants were included in the analysis. The demographic characteristics of the sample can be seen in Table 1. This study was reviewed and approved by the University of Bath Psychology Research Ethics Committee; reference number 20–171. The participants provided informed consent to participate online. As participants may have been concerned about disclosing information about their behaviour in relationships, the study was conducted anonymously.

**Table 1:** Demographic characteristics of the participants.

**Participant characteristics**		**(*n* = 149)**
Age (years), mean ± s.d	Range 18–77	34.28 ± 14.90
Gender, *n* (%)	Women (including transgender women)	118 (79.2)
	Men (including transgender men)	31 (20.8)
Ethnicity, *n* (%)	White: e.g., English/ Welsh/ Scottish/ Northern Irish/British/Irish	135 (90.6)
	Mixed/Multiple Ethnic Groups: e.g., White and Asian	4(2.7)
	Asian: e.g., Indian, Pakistani, Chinese, Japanese	3 (2)
	Arab/Any Other Ethnic Group	2 (1.3)
Sexual orientation, *n* (%)	Heterosexual	132 (88.6)
	Homosexual	6 (4)
	Bisexual	8 (5.4)
	Other	2 (1.3)
	Prefer not to say	1 (0.7)
Relationship status*, n* (%)	Married	45 (30.2)
	Widowed	1 (0.7)
	Divorced	2 (1.3)
	Separated	1 (0.7)
	In a long-term exclusive relationship (>2+ years)	43 (28.9)
	In an exclusive relationship (<2 years)	24 (16.1)
	In a causal relationship	6 (4)
	Single	27 (18.1)
Number of long-term relationships, *n* (%)	0	44 (29.5)
	1	61 (40.9)
	2	26 (17.4)
	3	12 (8.1)
	4+	6 (4)

The participants were recruited via an advert on the researcher's social media pages (Facebook, Instagram and LinkedIn), with a request that people share the advert on their own pages to broaden the recruitment scope rather than limit the sample to the researchers' immediate circle. The participants were offered the opportunity to enter their email address into a prize draw to win a £50 Amazon voucher as an incentive for their participation.

### Materials

A questionnaire battery consisting of four self-report measures, was built using Qualtrics. The participants were able to access the online questionnaire from any device with access to the internet, such as a phone, tablet or laptop.

Initially, the participants completed a demographics questionnaire to gather general characteristics of the participants such as age, gender, and relationship history. The participants were asked “Which gender do you identify as?” with the options Man (including transgender men), Women (including transgender women), Non-binary, Other, prefer not to say. However, every participant categorised themselves within either the Man (including transgender men) or Women (including transgender women) therefore only these categories were included in the subsequent analysis.

The first self-report measure was the Relationship Structures questionnaire (ECR-RS), which was used to measure adult attachment security providing an Insecure Anxious and Insecure Avoidant score (Fraley et al., 2011). This questionnaire consists of 9 items which require a response on a 7-point Likert scale to show how much the participants agree or disagree with each statement from 1 (strongly disagree) to 7 (strongly agree). It was only answered in relation to romantic partners, rather than across different types of relationships. This measure has been found to be reliable and consistent both internally, *α* > 0.80, and with other similar measures such as the ECR-R based on a sample of over 21,000 individuals (Fraley et al., 2000, 2011).

The second questionnaire measure was the 9-item Adult Disorganised Attachment scale (ADA), which was used to measure disorganised attachment in adult romantic relationships (Paetzold et al., 2015). For each item a response on a 7-point Likert scale is required to show how much the participants agree with each statement from 1 (strongly disagree) to 7 (strongly agree) is required. This measure has high internal consistency, α = 0.91, and high sampling adequacy (KMO = 0.91); each item has also been found to have high face validity (Paetzold et al., 2015).

The 29-item Buss Perry Aggression Questionnaire was used to measure controlling punitive behaviours; it contains four subscales, measuring physical aggression, verbal aggression, anger and hostility (Buss and Perry, 1992). The responses are measured via a 5-point Likert Scale to indicate how characteristic each of the statements is in describing the participant from 1 (extremely uncharacteristic of me) to 5 (extremely characteristic of me). Each subscale has been found to be stable over 7 months of testing, have moderate to high internal consistency and the scale also has sufficient construct validity (Harris, 1997).

Finally, the controlling caregiving and compulsive caregiving subscales from the 32-item Caregiving Questionnaire (CQ) were used to measure adults' approach to caregiving in relationships (Kunce and Shaver, 1994). These constructs were measured via a response of a 6-point Likert scale on which the participants indicated how descriptive each statement was of them ranging from 1 (not at all descriptive of me) to 6 (very descriptive of me). This measure has been found to be stable after a 1-month retest period, with each subscale having acceptable alpha reliability coefficients; *α* > 0.80 (Kunce and Shaver, 1994). The measure has also been found to have convergent validity with the Experiences in Close Relationships Questionnaire and to be reliable for same sex, as well as heterosexual couples (Brennan et al., 1998; Bouaziz et al., 2013).

### Design

The current study contained four self-report measures presented in an anonymous online questionnaire battery. For the first hypothesis, the independent variable was Disorganised attachment and the dependent variable was controlling caregiving behaviours. For the second and third hypotheses, the independent variables were Disorganised attachment and Insecure Avoidant attachment, respectively, and the dependent variable was controlling punitive behaviours. Hypotheses 2 and 3 were tested within one model. For the fourth hypothesis the independent variable was Insecure Anxious attachment and the dependent variable was compulsive caregiving behaviours. The order in which the questionnaires were presented to the participants was randomised to counterbalance any effects of order on the results. As there were multiple questionnaire measures within this study, a progress bar was included to encourage participants to complete the study.

### Procedure

The participants accessed the experiment from a link in the advertisement of the study. Initially they read an information sheet and completed a consent form before progressing onto the demographics' questionnaire. Following these initial stages, the four self-report measures were presented to participants. They were asked to answer every question, on a Likert scale, in respect to their current or most recent romantic relationship. The questionnaire generally took the participants around 15 minutes to complete. Having completed the questionnaire measures, the participants were debriefed and thanked. They were then given the opportunity to enter their email address into a prize draw to win a £50 Amazon voucher.

## Results

Three simultaneous multiple regressions were run to test each of the dependent variables: controlling caregiving, punitive behaviour and compulsive caregiving. Each model also controlled for the other attachment styles, gender and the participants long-term relationship history. The correlations between each variable can be seen in Appendix A in Supplementary Material. No outliers were detected, and no problems with collinearity were identified via the VIF value. However, visual inspection of the distribution of standardised residuals against the standardised predicted values, for each regression model, suggested that the models suffered from violations of the assumption of normality and homoscedasticity. Therefore, the ordinal least squared estimates are complimented with 95% percentile bootstrapped confidence intervals (2,000 resamples).

### Hypothesis One

Controlling caregiving (*M* = 20.93, *SD* = 17.04) was regressed onto Disorganised attachment (*M* = 37.11, *SD* = 6.98) to test the first hypothesis: participants with higher levels of Disorganised attachment would be more likely to engage in controlling caregiving behaviours in romantic relationships. As shown in Table 2, none of the variables accounted for a statistically significantly proportion of the unique variance in controlling caregiving.

**Table 2:** Summary of multiple regression analysis predicting controlling caregiving.

**Predictor variables**	***B***	***SE*_**B**_**	***β***	***sr***^***2***^	***p***
Gender	−0.48 [−3.29, 2.44]	1.56	−0.03	<0.01	0.760
Long-term relationship	0.77 [−2.08, 3.86]	1.42	0.05	<0.01	0.588
Insecure anxious attachment	0.32 [−0.12, 0.79]	0.20	0.15	0.02	0.115
Insecure avoidant attachment	−0.06 [−0.57, 0.39]	0.22	−0.03	<0.01	0.776
Disorganised attachment	0.09 [−0.09, 0.31]	0.10	0.08	<0.01	0.368
Overall model fit	*F*_(5, 148)_ = 1.15, *p* 0.34, *R*^2^ = 0.04 n.s.				

*Ninety-five percentile bootstrap confidence intervals for B (2,000 samples) are shown in brackets. Correlations were significant at the level 0.05*.

### Hypotheses Two and Three

Punitive behaviour (*M* = 67.36, *SD* = 7.63) was regressed onto Disorganised attachment (*M* = 37.11, *SD* = 6.98) and Insecure Avoidant attachment (*M* = 27.16, *SD* = 3.22) to test two hypotheses: participants with higher levels of Disorganised attachment or Insecure Avoidant attachment would be more likely to engage in controlling punitive behaviours in romantic relationships. As shown in Table 3, only Disorganised attachment and Insecure Anxious attachment accounted for a statistically significant proportion of the unique variance in punitive behaviours.

**Table 3:** Summary of multiple regression analysis predicting punitive behaviour.

**Predictor variables**	***B***	***SE***_**B**_	***β***	***sr***^***2***^	***p***
Gender	−6.16 [−12.09, −0.01]	3.20	−0.15	0.02	0.056
Long-term relationship	−2.39 [−8.19, 3.30]	2.93	−0.07	<0.01	0.417
Insecure anxious attachment	0.82 [0.3, 1.69]	0.41	0.17	0.02	0.048^*^
Insecure avoidant attachment	−0.36 [−1.22, 0.47]	0.45	−0.07	<0.01	0.425
Disorganised attachment	0.57 [0.20, 0.97]	0.20	0.24	0.05	0.005^**^
Overall model fit	*F*_(5,148)_ = 6.14, *p* < 0.01, *R*^2^ = 0.17						

Ninety-five percentile bootstrap confidence intervals for B (2,000 samples) are shown in brackets. Correlations that are significant at the 0.01 level are shown with ^**^ and those that are significant at the level 0.05 are shown with ^*^.

### Hypothesis Four

Compulsive caregiving (*M* = 26.76, *SD* = 7.04) was regressed onto Insecure Anxious attachment (*M* = 6.45, *SD* = 3.51) to test the fourth hypothesis: participants with a higher Insecure Anxious attachment style would be more likely to engage in compulsive caregiving behaviours in romantic relationships. As shown in Table 4, Insecure Anxious and Avoidant attachment accounted for a statistically significant proportion of the unique variance in compulsive caregiving.

**Table 4:** Summary of multiple regression analysis predicting compulsive caregiving.

**Predictor variables**	***B***	***SE***_**B**_	***β***	***sr***^***2***^	***p***
Gender	−2.03 [−4.77, 0.76]	1.35	−0.12	0.01	0.133
Long-term relationship	−1.61 [−3.94, 0.77]	1.23	−0.11	0.01	0.193
Disorganised attachment	0.05 [−0.11, 0.23]	0.09	0.05	<0.01	0.577
Insecure avoidant attachment	−0.40 [−0.87, 0.002]	0.19	−0.18	0.03	0.038^*^
Insecure anxious attachment	0.36 [−0.04, 0.78]	0.17	0.18	0.02	0.042^*^
Overall model fit	*F*_(5,148)_ = 5.01, *p* < 0.01, *R*^2^ = 0.15						

Ninety-five percentile bootstrap confidence intervals for B (2,000 samples) are shown in brackets. Correlations that are significant at the level 0.05 are shown with ^*^.

## Discussion

Controlling behaviours can cause difficulties within interpersonal relationships leaving people neglecting their own needs or the needs of their partner (Liotti, 2011). Therefore, the current study aimed to explore controlling behaviours and where they originate from, not to stigmatise individuals who rely on controlling behaviours but rather to understand them better and support healthy interpersonal relationships for individuals with insecure or disorganised attachment style. As it transpired, this focus was particularly relevant in light of the Covid-19 pandemic, as the importance of interpersonal relationships and support networks have become more apparent than ever before (Pietromonaco and Overall, 2020). Despite the fairly small effect sizes, the results revealed a significant positive relationship between disorganised attachment in adult romantic relationships and an engagement in controlling punitive behaviours. Additionally, participants higher in insecure anxious attachment were found to engage in more compulsive caregiving behaviours, whilst those higher in insecure avoidant attachment were found to engage in significantly less compulsive caregiving behaviours. An association was not found between disorganised attachment and controlling caregiving behaviours, nor insecure avoidant attachment style and controlling punitive behaviours.

### Disorganised Attachment and Controlling Caregiving

In contrast to the first hypothesis, there was no significant relationship found between disorganised attachment and controlling caregiving behaviours in adult romantic relationships, meaning that there was insufficient evidence to accept the first hypothesis. Unlike this study, many previous studies have found an association between disorganised attachment and engagement in controlling caregiving behaviours (Green and Goldwyn, 2002; Lyons-Ruth, 2007; Liotti, 2011, 2017). However, others have struggled to find a strong association (Bureau et al., 2009). In these studies, it was suggested that the lack of a relationship might be explained by the high attrition rate which meant that the most vulnerable children may have not completed the study, thus potentially impacting the results (Bureau et al., 2009). This could also have been the case for the current study.

To unpack this argument, it is important to examine the characteristics of the most vulnerable people. Whilst all insecure attachment styles have been associated with an increased risk for vulnerability and psychopathology, this is particularly true for those with a disorganised attachment style (Liotti and Gumley, 2008; Fearon et al., 2010). Previous studies have shown that individuals with a disorganised inverted attachment style, where the infant has cared for their guardian rather than the guardian caring for them, most often engage in controlling caregiving behaviours (Liotti, 2011; Hollidge and Hollidge, 2016). In such instances the guardian is often helpless or frightened and may seek comfort and protection from their child (Moss et al., 2005; Solomon and George, 2011). Disorganised attachment and caregiving role reversal can occur when caregivers experience violence and domestic abuse, drug or alcohol dependency or mental health difficulties that leave the infant vulnerable (Brandon et al., 2008). Therefore, due to the adverse childhood experiences and trauma, people with a disorganised inverted attachment style are generally more likely to engage in controlling caregiving behaviours and develop a mental health condition. Since these people represent the most vulnerable population, it reasonable to expect that such individuals may not be adequately represented in this study's non-clinical sample. Therefore, perhaps this study did not recruit participants from the type of population in which one is most likely to find an association between controlling caregiving and disorganised attachment; in turn, this could explain the null finding in the current study. As personality disorders are extremes of normal behaviours that exist across the population, such as engagement in controlling behaviours, this study looked at the prevalence of such behaviours across the general population rather than using a clinical sample. However, future research should recruit a clinical population to explore whether the relationship exists in this adult population, given its prevalence amongst children (Liotti, 2011, 2017).

### Disorganised Attachment and Controlling Punitive Behaviour

People with higher levels of disorganised attachment were significantly more likely to engage in controlling punitive behaviour, as measured by the Buss Perry Aggression Scale (Buss and Perry, 1992). On this basis, there was sufficient evidence to accept the second hypothesis, thus supporting a wealth of previous research that has found an association between the disorganised attachment style in children and adolescents and engagement in controlling punitive behaviour to organise interactions with their caregiver (Main and Cassidy, 1988; Cassidy and Marvin, 1992; van IJzendoorn et al., 1995; Green and Goldwyn, 2002; Moss et al., 2004; Lyons-Ruth, 2007; Lyons-Ruth and Jacobvitz, 2008; Liotti, 2011, 2017).

This result suggests that engagement in such behaviours may stabilise into adulthood, as some studies have predicted (Brennan and Shaver, 1998; Moss et al., 2005; Solomon, 2018). Engagement in controlling punitive behaviours could cause difficulties in romantic relationships such as for conflict resolution and could even have legal ramifications (Brennan and Shaver, 1998; Shi, 2003; Paetzold et al., 2015; Solomon, 2018). Coercive control, for example, which is defined as controlling behaviour towards another to make them subordinate or dependent through threats, humiliation or intimidation has recently become a criminal offence under section 76 of the Serious Crime Act, 2015, in the context of two people who are personally connected (Hamberger et al., 2017; Walby and Towers, 2018; Stark and Hester, 2019; Storey, 2019). As the law now recognises the more psychological types of control within adult relationships, it was deemed important for this study to follow suit to help to gain a deeper understanding of these behaviours which can greatly impact vulnerable individuals' ability to function well in relationships.

It is also important to consider the applied implications of the current findings for clinical conditions like borderline personality disorder (BPD). Whilst many studies have shown that adverse childhood experiences and disorganised attachment are risk factors for BPD, the mechanisms linking childhood experiences and the development of BPD in adulthood have not yet been fully identified (Leichsenring et al., 2011). Disorganised attachment is generally the result of adverse early childhood experiences, such as trauma or neglect (Brandon et al., 2008). Therefore, although the current study cannot explain the complex pathway from disorganised attachment to a later diagnosis of BPD, it could inform future studies seeking to explore the link between adverse childhood experiences, attachment, and psychopathology further.

### Insecure Avoidant Attachment and Controlling Punitive Behaviour

A significant relationship was not found between insecure avoidant attachment and controlling punitive behaviours; hence there was insufficient evidence to accept the third hypothesis. It was expected that people who scored higher in insecure avoidant attachment would engage in more controlling punitive behaviours, since insecure avoidant individuals have been found to display more dominance-seeking behaviour, use more antisocial behaviour and present with more externalising problems (Troy and Sroufe, 1987; Westen et al., 2006; Yip et al., 2018). As controlling punitive behaviours are also characterised by hostile and angry (antisocial) behaviours that are used to compete for social dominance, a positive relationship with avoidant attachment was expected (Hawley et al., 2009). However, the results revealed that the expected relationship was not found.

Previous research has documented inconsistent findings regarding avoidant attachment style; some studies suggests a greater association with externalising problems and aggressive antisocial behaviour whilst others have found that highly avoidant adults use defence mechanisms such as withdrawal and distance-seeking within relationships in order to deactivate their attachment system (Simpson et al., 1992; Creasey et al., 1999; Mikulincer and Shaver, 2003; Paetzold et al., 2015). This could explain the null finding for this hypothesis; insecure avoidant individuals may avoid conflict in romantic relationships and be more likely to engage in passive aggressive punitive behaviours rather than those measured by the Buss Perry Aggression Scale. It may be the case that an alternative measure would be more suitable for exploring controlling punitive behaviours among people with an insecure avoidant attachment; future research may wish to investigate alternative presentations of the insecure avoidant attachment style in adult romantic relationships.

### Insecure Anxious Attachment and Compulsive Caregiving

Participants with higher levels of insecure anxious attachment reported engaging in more compulsive caregiving strategies; hence there was sufficient evidence to accept the fourth hypothesis. This finding contributes to attachment literature, as current studies considering the use of controlling behaviours have generally focused on disorganised attachment rather than all types of insecure attachment. The compulsive need to be needed and involved in a partners' problems is also a common characteristic of dependent personality disorder which has been linked with insecure anxious attachment in previous literature (Sinha and Sharan, 2007). Hence this result could help explain a mechanism linking insecure attachment style and personality disorders characterised by neediness and dependency.

This hypothesis was further supported by the finding that participants higher in insecure avoidant attachment were significantly less likely to engage in compulsive caregiving. This finding supports previous research, which states that highly avoidant adults seek distance when their partner is distressed and are less inclined to become involved in their problems (Groh et al., 2012; Paetzold et al., 2015).

These findings have implications for understanding interpersonal difficulties that insecurely attached adults can face. Insecure anxious adults frequently report resentment in relationships as they feel their own needs are not adequately met and avoidant adults seem to have less desire to support their partner when distressed or become entangled in their problems (Kunce and Shaver, 1994; Groh et al., 2012). Identifying these patterns of behaviour in relationships and the individuals who are at a higher risk of engaging in them, could help inform future interventions in the interest of promoting healthy interpersonal relationships.

## Conclusion

This research has three main conclusions regarding the relationship between attachment style and controlling behaviours in adult romantic relationships. First, adults with a more disorganised attachment style in romantic relationships are more likely to engage in controlling punitive behaviours. Second, people with an insecure-anxious attachment style are more likely to engage in compulsive caregiving behaviours and in this way, become overinvolved in their partners' problems. Third, people with a more avoidant attachment style were less likely to engage in compulsive caregiving, indicating a tendency to withdraw from supporting their partners when in need. These tendencies can harm individual's ability to function well in interpersonal relationships. Furthermore, the associations with attachment style may help to explain some of the mechanisms that contribute to the development of personality disorders. In the future, this knowledge could aid the understanding of borderline personality disorder since the trajectory from attachment style to a later BDP diagnosis is not yet fully understood, with debate in existing literatures regarding which attachment style is most associated with BDP (Main et al., 1985; Selby et al., 2008; Levy et al., 2015; Beeney et al., 2017).

It is also important to note the limitations of the current study; the effect sizes were fairly small, the method for measuring the gender variable was simple and a large proportion of the sample identified as women which could have impacted on the results, as previous studies have found gender differences in caregiving and controlling behaviours (Fawson, 2015; O'Campo et al., 2017; Levendosky et al., 2018). Additionally, this study did not consider whether the participants had children which could have been a confounding variable, as being a parent could impact caregiving behaviours within relationships, future studies may wish to explore this further. Additionally, future research may wish to consider factors that mediate the relationship between attachment style and controlling behaviours, such as affect regulation which is well-documented to contribute to the development of personality disorders. Previous studies have found an association between an inability to regulate emotions effectively and disorganised attachment (Townshend and Caltabiano, 2019). Studies have suggested that trauma, coercive parenting, and a lack of maternal sensitivity can impact an infant's development of affect regulation, which in turn can affect the way traumatised people react when frustrated, leading to an increased in antisocial behaviour and aggression (Van Egeren et al., 2001; Dennis, 2006; Kim, 2010). Unstable affect regulation could be an underlying explanation for the use of aggression and hostile behaviours in disorganised individuals which might relate to the controlling punitive behaviours measured in the current study (Kim, 2010). Future research could explore whether there is a relationship between affect regulation and engagement in controlling behaviours. In turn, this could explain the use of controlling punitive behaviours among people with a disorganised attachment style, as found in the current study.

## Data Availability Statement

The raw data supporting the conclusions of this article will be made available by the authors, without undue reservation.

## Ethics Statement

The studies involving human participants were reviewed and approved by The University of Bath Psychology Research Ethics Committee. Written informed consent for participation was not required for this study in accordance with the national legislation and the institutional requirements.

## Author Contributions

MG developed the idea for the overall study and the research questioned, also designed the study and gained the appropriate ethical approval from the Department of Psychology at the University of Bath, responsible for recruiting participants according to the inclusion criteria, collecting, and all of the data, and reporting the results, and reviewed the relevant literature within the field and wrote the research paper. RB contributed guidance on the topic area and research questions followed by advise regarding the interpretation of the data and also provided feedback and advice on a draft copy of the report as well as some amendments. All authors contributed to the article and approved the submitted version.

## Conflict of Interest

The authors declare that the research was conducted in the absence of any commercial or financial relationships that could be construed as a potential conflict of interest.
