# Supplementary figures and images for: Exploring How UK Adults' Attachment Style in Romantic Relationships Affects Engagement in Controlling Behaviours
Source: Front Psychol. 2021 Jun 30;12:649868. doi: 10.3389/fpsyg.2021.649868 (PMC8279754; doi:10.3389/fpsyg.2021.649868)

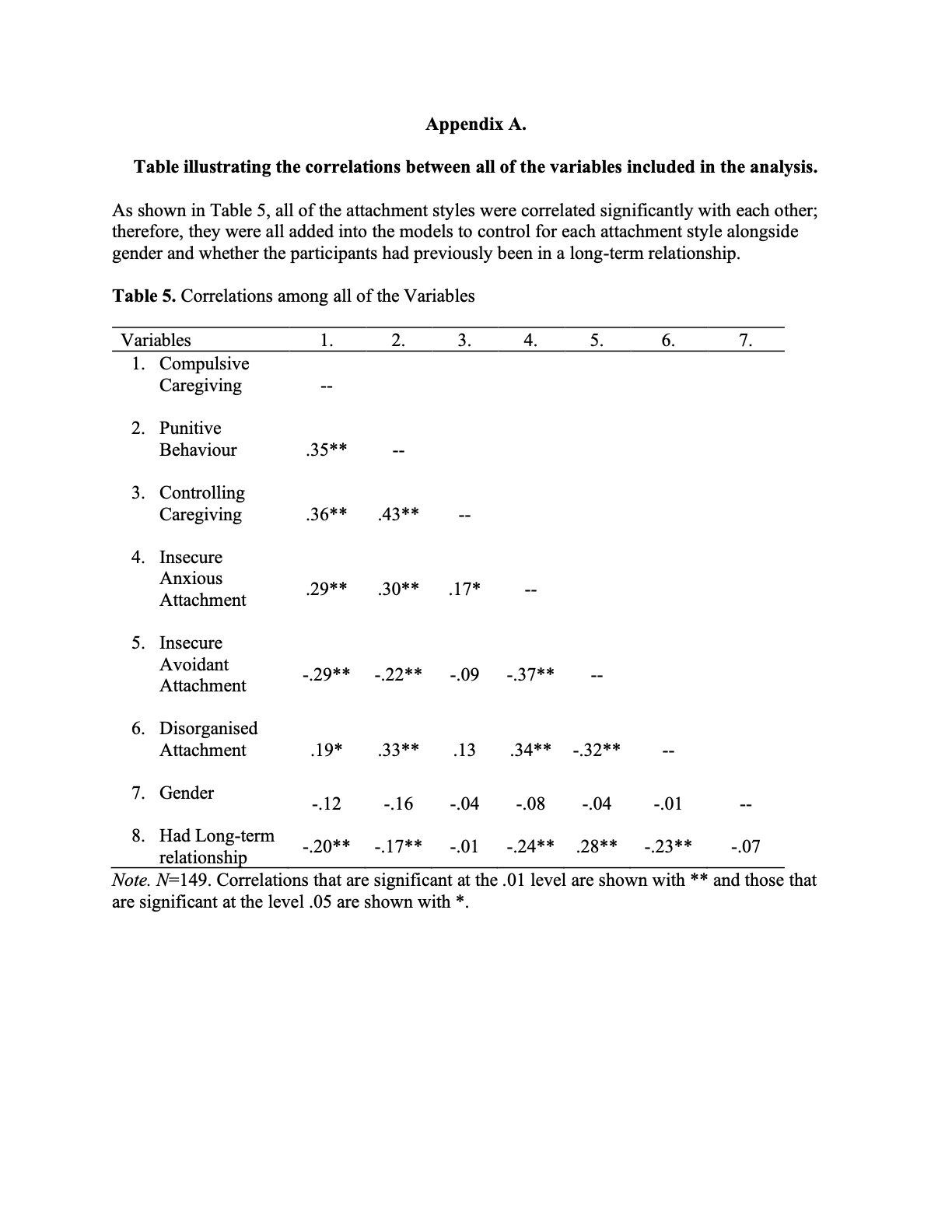

Supplement: Supplementary file 1 [file Image_1.JPEG]
